# Large Language Models in Clinical Trial Recruitment: Sociotechnical and Economic Framework Development Study

**DOI:** 10.2196/95899

**Published:** 2026-05-20

**Authors:** Qian Qian

**Affiliations:** 1 Department of Industrial and Systems Engineering, Faculty of Engineering, Hong Kong Polytechnic University, Room EF625, Core F, Main Campus, Hung Hom, Kowloon, Hong Kong, China (Hong Kong), 86 15021400019, 86 15021400019

**Keywords:** clinical trials, artificial intelligence, large language models, patient selection, sociotechnical systems, transaction cost economics, human-AI collaboration, clinical AI, governance

## Abstract

**Background:**

Large language models (LLMs) have shown substantial promise in patient-trial matching, but most published studies still evaluate the performance under controlled technical conditions rather than within real recruitment workflows. Less is known about how LLM-enabled clinical artificial intelligence (AI) systems should be embedded into organizational settings where privacy constraints, human oversight, patient-facing concerns, and governance costs shape deployment outcomes.

**Objective:**

This study develops a theory-grounded conceptual framework for analyzing how LLM-enabled clinical AI can be integrated into clinical trial recruitment workflows and how such integration affects operational, governance, and economic outcomes.

**Methods:**

Using structured conceptual analysis and targeted evidence synthesis, the study draws on recent literature on LLM-based patient matching, human-AI collaboration in clinical settings, and clinical AI governance. Sociotechnical systems theory and transaction cost economics are integrated to build the LLM-Embedded Clinical Recruitment Architecture (LECRA) and to derive 6 testable propositions.

**Results:**

LECRA conceptualizes recruitment as a closed-loop sociotechnical and economic system, spanning data complexity, model configuration and processing, human-AI collaboration, and economic and governance consequences. The revised framework identifies privacy constraints, hallucination risk, bias, patient trust, oversight intensity, and regulatory validation costs as key moderators of performance. It also reframes recruitment performance as a multidimensional construct and outlines an empirical roadmap for future testing.

**Conclusions:**

LECRA offers a more deployment-sensitive account of when LLM-enabled recruitment is likely to create value and when the benefits may be offset by coordination, compliance, or trust-related frictions. This framework is intended to support future empirical studies and more realistic implementation decisions rather than to claim validated superiority of LLM-assisted recruitment.

## Introduction

### The Recruitment Bottleneck

Clinical trial recruitment is widely recognized as one of the most persistent bottlenecks in pharmaceutical research. A 30-year retrospective analysis of trial accrual at MD Anderson Cancer Center found that slow enrollment—defined as fewer than 2 participants per year—was not an occasional setback but a systemic pattern, driven by structural factors, including inaccurate accrual projections, overly restrictive eligibility criteria, and limited coordination across participating sites [1]. The costs of recruitment delays are substantial: industry estimates suggest that each day of delayed enrollment can cost sponsors between US $600,000 and US $8 million in lost revenue, depending on the therapeutic area.

### Large Language Model Capabilities in Patient-Trial Matching

Recent advances in large language models (LLMs) have generated considerable interest in their application to this problem. The TrialGPT framework demonstrated that a zero-shot LLM pipeline could recall over 90% of relevant clinical trials while reducing the candidate search space to less than 6% of the total corpus [2]. Multistage retrieval pipelines that combine conventional information retrieval with LLM-based reranking have outperformed either approach in isolation on standardized benchmarks [3]. Prompt-based approaches have shown promise in extracting eligibility-relevant information from unstructured clinical notes [4], and hybrid frameworks integrating LLMs with knowledge graphs have demonstrated potential for transforming passive recruitment into an active, artificial intelligence (AI)-assisted process [5]. A recent scoping review covering LLM applications in patient-trial matching between late 2022 and the end of 2024 confirmed that the field is expanding rapidly, albeit with significant methodological heterogeneity [6].

The application of natural language processing to clinical trial eligibility matching has a history that predates the current generation of LLMs. A systematic review of natural language processing methodologies in this domain documents the progression from rule-based extraction and ontology-driven systems through statistical machine learning to the transformer architectures that underpin contemporary LLMs [7]. Earlier approaches were typically constrained by their dependence on structured data fields and predefined terminologies, which limited their capacity to interpret the nuanced, context-dependent eligibility criteria common in real trial protocols.

In more applied settings, Beattie et al [8] reported that GPT-based automation of patient screening reduced staff workload while maintaining matching quality acceptable for routine use, though the authors emphasized the continued necessity of human oversight for ambiguous cases. Rahmanian et al [4] explored prompt-based cohort selection from medical notes annotated with SNOMED CT (Systematized Nomenclature of Medicine—Clinical Terms) and found that performance varied appreciably with criteria complexity. Beyond matching, Gao et al [9] demonstrated that LLMs could generate trial-specific educational materials for potential participants, and Jin et al [10] investigated LLM assistance in the upstream task of randomized controlled trial design.

Taken together, these studies paint a picture of rapid technical progress. However, the evidence base has an important limitation that Chen et al [6] articulate clearly in their scoping review: almost all evaluations are conducted in controlled settings, and evidence from real-world operational deployment remains scarce.

### Human-AI Collaboration in Clinical Settings

A parallel stream of literature has examined how humans and AI systems work together in clinical decision-making. The central insight of this literature is that AI’s impact on clinical outcomes depends not only on the model’s standalone accuracy but also on the dynamics of the human-AI team.

Liu et al [11] conducted a meta-analysis of 52 empirical studies of human-AI teaming in health care and reported 2 key findings. First, while AI generally augmented clinician performance, full complementarity—where the human-AI team exceeded both individual baselines—was achieved less often than expected. Second, the teaming mode mattered: simultaneous review (where clinicians considered AI outputs alongside their own assessment) outperformed sequential hand-off. In the specific context of trial recruitment, Guido et al [12] provided one of the few randomized results, showing that human-AI teams performing oncology prescreening matched the accuracy of manual chart review while substantially reducing processing time.

Qualitative research adds a further dimension. Hassan et al [13], through semistructured interviews with clinical professionals, found that explainability mechanisms were regarded as essential for sustaining trust, particularly when clinician judgment diverged from AI recommendations. The ability to understand why the system reached a particular conclusion—and to override it—was viewed as a nonnegotiable safety feature. These findings suggest that the design of human-AI interaction, not merely the accuracy of the underlying model, is a primary determinant of system-level performance.

### Organizational and Economic Perspectives

A smaller but growing body of work addresses the broader organizational and economic dimensions of AI adoption in health care. Khashu [14] observed that, despite substantial investment, real-world AI adoption in clinical settings remains limited and attributed this partly to the tendency of existing trustworthy-AI frameworks to emphasize technical model properties at the expense of operationalization in actual workflows. Li et al [15] proposed the TRIAD framework—addressing trustworthy governance, real-world clinical value, and integrated adaptive deployment—as a more comprehensive approach, though without specifying the economic mechanisms through which AI generates organizational values.

The economic dimension is notably absent from most studies of LLMs in clinical trial recruitment. While technical papers frequently report reductions in processing time or improvements in matching recall, these metrics are rarely translated into cost estimates, revenue implications, or return-on-investment calculations. This omission is consequential: without credible economic evidence, the case for organizational investment in LLM-based recruitment systems rests on technical promise rather than the demonstrated value.

### Research Gap and Objectives

The literature reviewed above reveals a field characterized by rapid technical progress but limited attention to workflow-level integration, governance burden, and economic consequences. Three gaps are particularly salient. First, most recruitment studies remain focused on component-level matching performance rather than on how LLM outputs are embedded into real organizational workflows. Second, the technical literature on patient-trial matching remains weakly connected to the literature on human-AI collaboration, despite the fact that recruitment decisions are typically mediated by human review, escalation, and accountability structures. Third, the organizational and economic consequences of LLM adoption, including privacy controls, validation costs, and oversight burdens, remain under-specified.

The present analysis addresses these gaps by developing the LLM-Embedded Clinical Recruitment Architecture (LECRA), a theory-grounded conceptual framework that explains how data complexity, model configuration, human oversight, and governance-cost structures jointly shape recruitment outcomes. The paper makes 3 contributions. First, it offers a recruitment-specific sociotechnical and economic framework rather than a generic account of AI adoption in health care. Second, it explains how technical performance, collaboration design, and economic consequences are linked through identifiable mechanisms and boundary conditions. Third, it derives 6 testable propositions and an empirical evaluation roadmap that can guide future studies of LLM-enabled recruitment in real organizational settings. Accordingly, the aim of this paper is to develop a theory-grounded conceptual framework for analyzing how LLM-enabled clinical AI systems can be integrated into clinical trial recruitment workflows and to derive propositions and an evaluation roadmap for assessing their operational, governance, and economic consequences.

## Methods

### Study Design

This study adopts a conceptual theory-building design rather than an empirical design. It uses structured conceptual analysis and targeted evidence synthesis to integrate findings from 3 adjacent streams: LLM-based patient-trial matching, human-AI collaboration in clinical settings, and governance or organizational studies relevant to clinical AI deployment. The objective is explanation and proposition development, not causal estimation. Therefore, the paper treats LECRA as the principal conceptual output of the study and the propositions as analytically derived claims that require future empirical testing.

Evidence was identified through targeted searches of PubMed, Scopus, and Google Scholar using combinations of terms such as “large language model,” “clinical trial recruitment,” “patient matching,” “human-AI collaboration,” “AI governance,” and related workflow terms. Recent LLM-specific evidence from 2022 to 2026 was prioritized, while foundational sociotechnical systems (STS) and transaction cost economics (TCE) sources were retained as theoretical anchors. The inclusion criteria required that sources contribute directly to one or more of the following: recruitment workflow design, model-assisted patient matching, collaboration structure, governance and explainability, or economic implications of deployment. The exclusion criteria were studies or commentaries focused only on unrelated administrative AI use, nonclinical matching tasks, purely technical benchmark optimization without recruitment relevance, or opinion commentary without a clear conceptual, empirical, or implementation contribution. The practical bounds of the search were topical and conceptual rather than exhaustive; selection was limited to literature capable of informing LECRA constructs, mechanisms, or boundary conditions, and additional sources were not retained if they did not alter the framework logic or proposition development. This was not designed as a formal systematic review; rather, it was a structured conceptual synthesis. When identified, divergent or cautionary evidence was considered to reduce confirmation bias and avoid overstating consensus.

### Sociotechnical Systems Theory

The STS theory, originating in the work of Trist and Bamforth [16] and subsequently elaborated by Emery and Trist [17] and later systems-engineering accounts of sociotechnical design [18], holds that complex work systems contain interdependent technical and social subsystems. Its central principle of joint optimization argues that system-level performance cannot be maximized by optimizing tools, workflows, or human roles in isolation. This insight is especially relevant to clinical trial recruitment, where LLM outputs must be interpreted, checked, escalated, and acted upon within regulated workflows rather than consumed as stand-alone predictions.

Applied to recruitment, the STS theory implies that technical capability alone is insufficient. A model that improves matching recall may still degrade system performance if outputs are difficult to verify, poorly aligned with reviewer responsibilities, or trusted too much or too little. Therefore, STS provides the logic for analyzing oversight intensity, explanation needs, discrepancy resolution, and patient-facing trust as endogenous design choices rather than downstream implementation details.

### Transaction Cost Economics

Transaction cost economics, as developed by Williamson [19,20], complements STS by foregrounding the costs of organizing, verifying, and monitoring transactions. In recruitment workflows, these costs arise not only from information processing but also from eligibility interpretation, coordination among clinical and operational actors, monitoring, documentation, and compliance review. Therefore, clinical trial recruitment involves more than a matching problem; it is also a recurring coordination problem under uncertainty.

LLM-enabled systems may reduce some transaction costs by automating parts of information extraction and preliminary matching, but they may also introduce new costs related to validation, auditability, model governance, and privacy control. This distinction is especially important in health care, where scale economies are determined not only by computing efficiency but also by how regulatory and organizational overhead is distributed across cases. Therefore, TCE helps explain why technically promising systems can still produce weak or even negative net value in real-world settings.

The TCE lens also clarifies why asset specificity matters for recruitment AI. Domain-adapted local models, institution-specific retrieval pipelines, and locally governed screening tools may offer stronger contextual fit and privacy control, but they also involve higher fixed costs, greater specificity to local data structures, and lower substitutability across organizations. Generic cloud APIs, by contrast, may be less asset-specific and more easily substituted, but they can create different governance concerns around data transfer, vendor dependence, auditability, and alignment with local eligibility interpretation. The cloud-versus-local distinction in layer 2 should therefore be understood not only as a technical architecture choice but also as a transaction-cost choice involving specificity, substitutability, and governance overhead.

### Integrating the 2 Perspectives

The STS theory and TCE illuminate different but complementary aspects of the problem. STS explains why the same model can perform differently under different collaboration designs because human review, explanation, escalation, and trust calibration shape whether technical capability is translated into usable workflow output. TCE explains why the economic consequences of those same designs depend on uncertainty, monitoring burden, and the fixed and variable costs of deployment.

Their integration is therefore not merely additive. LECRA uses STS to model how data, models, and human actors must be jointly configured, and it uses TCE to explain why those configurations generate different coordination costs, oversight burdens, and scale effects. The propositions were derived by tracing how each framework dimension alters expected recruitment performance, what mechanism is proposed to explain that relationship, and under what conditions that relationship is likely to weaken or reverse.

### Ethical Considerations

This study is a conceptual analysis based exclusively on published literature and theory integration. It did not involve human participants, intervention delivery, primary data collection, or access to identifiable patient-level information; accordingly, institutional ethics review was not required for this paper. No informed consent was required because no participant data were collected or analyzed. No protected health information or confidential clinical records were used, no participant compensation was applied, and no identifiable participant images were included in the paper or supplementary materials. Future empirical applications of LECRA should undergo ethics and privacy review in accordance with local institutional and regulatory requirements.

## Results

### The LECRA Framework

**Figure 1.**
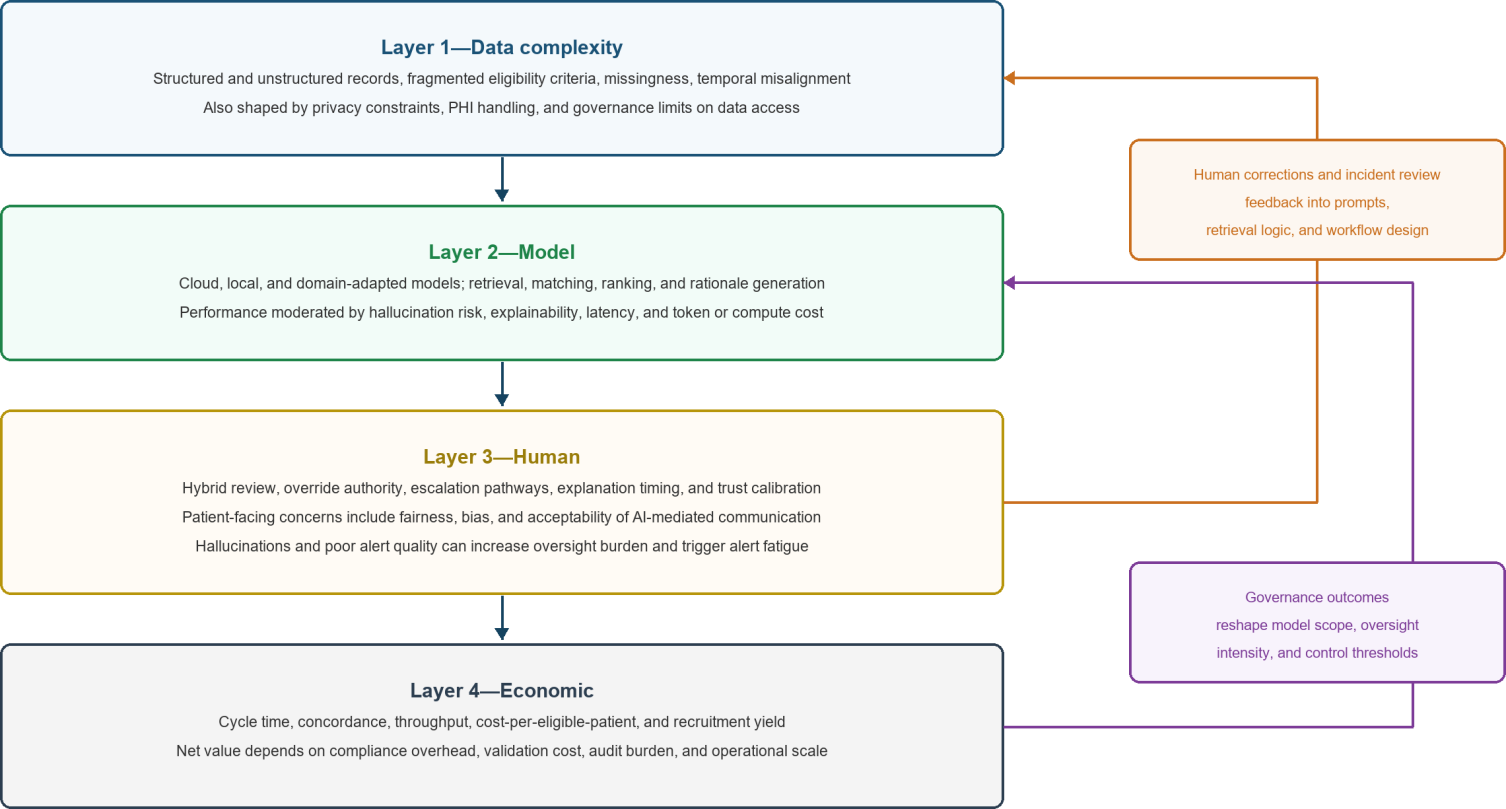
LECRA as a closed-loop sociotechnical and economic framework for clinical trial recruitment. The proposed model links data complexity, model configuration and processing, human-AI collaboration, and economic and governance outcomes. Forward arrows show the main causal flow from data conditions to model use, collaboration design, and economic consequences. Feedback loops indicate how reviewer corrections, incident reviews, and governance outcomes can inform prompt refinement, retrieval configuration, workflow redesign, and oversight thresholds over time. AI: artificial intelligence; LECRA: LLM-Embedded Clinical Recruitment Architecture; LLM: large language model; PHI: protected health information; STS: sociotechnical systems theory; TCE: transaction cost economics.

Building on the theoretical foundations outlined above, the LECRA is proposed as a 4-layer conceptual model with explicit feedback loops. Rather than treating recruitment as a simple pipeline from data to match output, LECRA conceptualizes it as a sociotechnical transaction system in which data complexity, model configuration, human oversight, and governance-economic consequences interact dynamically over time. Figure 1 presents the revised logic of the framework.

#### Layer 1: Data Complexity

The first layer captures the data environment in which recruitment decisions are made. Clinical trial matching depends on combining structured electronic records, unstructured clinical notes, laboratory summaries, trial protocols, and exclusion criteria written in natural language. These inputs differ in completeness, temporal alignment, terminology, and interpretability. In many settings, the problem is not merely data heterogeneity but the coexistence of missing, delayed, or partially contradictory information.

In TCE terms, these conditions increase transaction uncertainty and therefore raise the costs of verification. In clinical settings, data complexity also has a governance dimension: fragmented or sensitive records can trigger stricter access controls, additional deidentification work, and more expensive review procedures. Therefore, LECRA treats data complexity as both a technical constraint and a compliance-relevant moderator. Under this logic, high complexity may increase the value of LLM-enabled reasoning in some cases while simultaneously increasing privacy, audit, and oversight burdens that limit straightforward automation [4,5].

Modern clinical informatics standards may moderate this layer. Standardized structures such as Health Level Seven Fast Healthcare Interoperability Resources (HL7 FHIR) or the Observational Medical Outcomes Partnership (OMOP) Common Data Model can improve structured exchange, semantic consistency, and traceability across systems, whereas weak interoperability can increase extraction, mapping, and verification burden before LLM-enabled matching can be used reliably in operational settings. Therefore, LECRA treats interoperability not as a background technical detail but as a condition that shapes the feasibility and reliability of the data-embedding layer.

#### Layer 2: LLM Processing

The second layer represents model configuration and processing. Here, LECRA shifts away from treating LLMs as a monolithic technology. Deployment choices may involve commercial cloud models, private or local models, retrieval-augmented systems, or more domain-adapted configurations. These architectural choices shape latency, privacy exposure, explainability options, update cadence, and cost structure and, therefore, moderate both technical performance and deployment viability.

LLMs can reduce screening effort by extracting patient attributes from unstructured text, interpreting eligibility language, and producing preliminary rationales. However, technical capability remains bounded by hallucination, brittle reasoning under unusual documentation patterns, and uneven calibration across case types. Greater case complexity may also increase compute or token costs rather than reduce marginal costs. LECRA therefore treats uncertainty communication, rationale transparency, and architecture choice as design variables rather than optional enhancements. Technical performance is necessary but not sufficient; what matters is whether model outputs remain interpretable and governable within the workflow [2,3,8].

This architectural choice also has an economic interpretation. A locally adapted model or retrieval system may become a specific organizational asset because it is tuned to local documentation practices, trial portfolios, privacy requirements, and reviewer routines. Such specificity can improve contextual performance, but it makes the system more expensive to develop, maintain, and transfer. A generic cloud model may be cheaper to access and easier to replace, but its lower specificity may increase the need for additional verification, prompt engineering, or governance controls in sensitive recruitment contexts.

#### Layer 3: Human-AI Collaboration

The third layer operationalizes the STS principle of joint optimization. In clinical trial recruitment, LLM outputs are rarely actionable without human review because cases differ in ambiguity, eligibility criteria require interpretation, and accountability remains organizationally and professionally allocated. Therefore, the central design question is not whether humans should remain involved but how oversight intensity, override authority, explanation timing, and escalation logic should be configured so that technical capability improves rather than destabilizes workflow performance.

This layer also introduces risks that are underappreciated in benchmark-focused studies. Hallucinated rationales can increase reviewer burden, and repeated low-quality alerts can contribute to alert fatigue or defensive overchecking. Historical documentation bias may skew downstream recommendations, thereby affecting equity across patient groups. Patient involvement should also be represented carefully. In many recruitment service workflows, patients are contacted by medical recruitment specialists who clarify clinical histories, request missing documentation, and coordinate review by medical teams or clinical research coordinators. Therefore, patient-facing AI is not necessarily the main recruitment pathway. However, where locally permitted and governed by consent, privacy, and clinical review requirements, LLM-enabled systems can support an optional intake channel in which patients describe their condition through a conversational interface or upload heterogeneous materials, such as referral notes, laboratory reports, imaging files, discharge summaries, or photographs of medical documents. The system may then classify materials, extract trial-relevant information, generate a structured preliminary summary, and identify potentially relevant trial opportunities. These outputs should be treated as decision-support artifacts for recruitment specialists and clinical research coordinators, not as autonomous eligibility determinations. LECRA therefore treats collaboration design as the key mediator through which technical outputs become operational and social outcomes [11-13].

#### Layer 4: Economic Impact

The fourth layer captures economic and governance consequences. Improvements in screening speed or matching consistency may reduce labor requirements, narrow candidate pools more efficiently, and increase throughput. However, the relevant organizational question is not whether some operational metrics improve in isolation; it is whether those gains exceed the fixed and recurring costs introduced by deployment.

These costs may include workflow redesign, staff retraining, model integration, incident review procedures, audit logging, privacy safeguards, and regulatory validation obligations. For clinical AI, fixed costs associated with validation, good clinical practice–aligned review, or software classification can materially reduce or delay scale benefits. LECRA therefore qualifies the original scale economy argument: larger transaction volumes can improve the economics of deployment only when governance overhead remains proportionate and when marginal compute, monitoring, and compliance costs do not escalate faster than operational gains [2,12,15].

#### Interlayer Dynamics

The 4 layers are analytically distinct but dynamically interdependent. Data complexity determines what kind of model configuration is feasible. Model outputs are then filtered through the collaboration structure, which determines how uncertainty is communicated, challenged, or escalated. Operational effects feed into economic and governance outcomes, but these outcomes in turn influence subsequent model use, oversight thresholds, and workflow redesign decisions.

For this reason, LECRA is not a one-way pipeline. Reviewer corrections may be used to refine prompts, retrieval settings, or local knowledge resources. Incident review may lead organizations to tighten override rules, add new checkpoints, or narrow the scope of automation. Conversely, governance structures that are too weak may create short-run speed gains but degrade trust and increase downstream correction costs. These feedback loops are central to understanding why apparently similar systems can diverge over time in both operational and economic performance.

### Propositions

The revised framework generates 6 propositions regarding the conditions under which LLM integration is likely to improve recruitment performance. Here, recruitment performance is treated as a multidimensional construct rather than a single efficiency metric. Relevant dependent variables may include screening cycle time, eligibility concordance, enrollment yield, consent conversion, reviewer oversight burden, cost-per-eligible-patient, and equity-sensitive indicators where appropriate. Each proposition therefore specifies a focal relationship, a mechanism, and a boundary condition that future studies can examine empirically.

#### Proposition 1

Proposition 1 (data complexity → conditional LLM advantage): The performance advantage of LLM-enabled processing over rule-based approaches is likely to increase with data complexity when digital records remain sufficiently accessible for model-assisted interpretation.

Derivation: As uncertainty and interpretive burden rise, the value of flexible language-based processing increases relative to rigid rules. However, this advantage weakens when data fragmentation, access restrictions, or documentation gaps become so severe that neither the model nor the reviewer can reliably reconstruct the relevant clinical context [2,4,5]. Candidate outcomes include cycle time, criterion-level concordance, and disagreement rates across case-complexity tiers.

#### Proposition 2

Proposition 2 (model configuration → net information-processing gain): LLM-enabled systems are more likely to reduce the marginal information-processing burden of recruitment when model architecture, retrieval design, and privacy controls are aligned with the complexity and sensitivity of the data environment.

Derivation: Technical capability alone does not determine net gain. Cloud-based, local, and retrieval-augmented configurations differ in latency, auditability, privacy exposure, and compute cost. In high-complexity cases, token-intensive or weakly governed configurations may increase rather than decrease marginal processing cost. Observable implications include reviewer handling time, escalation frequency, compute or operating cost per case, and privacy-related workflow delay.

#### Proposition 3

Proposition 3 (joint optimization → hybrid superiority): Hybrid human-AI workflows are expected to outperform fully manual and fully automated workflows when model outputs are accompanied by auditable rationales, and human reviewers retain documented override authority.

Derivation: The STS theory predicts that neither technical capability nor human labor alone will maximize system performance in ambiguous, regulated settings. Hybrid systems become advantageous when reviewers can verify outputs efficiently rather than merely duplicate model work. This expectation weakens when rationales are opaque or when oversight roles are poorly specified [11-13]. Candidate outcomes include cycle time, eligibility concordance, and reviewer confidence or trust calibration indicators.

#### Proposition 4

Proposition 4 (automation risk → oversight burden): The operational gains from higher automation are expected to weaken or reverse in recruitment contexts characterized by high ambiguity, hallucination sensitivity, or sustained reviewer alert fatigue.

Derivation: Fully automated or weakly supervised workflows may generate fast preliminary outputs while shifting hidden verification and correction burdens downstream. When cases are highly ambiguous, hallucinated rationales or poorly calibrated alerts can erode trust and trigger maximum oversight intensity. Relevant outcomes include correction burden, disagreement resolution time, override rate, and safety-related incident review volume.

#### Proposition 5

Proposition 5 (scale → conditional economic benefit): The economic benefits of LLM integration are expected to increase with operational scale only when additional governance, validation, and compliance costs remain below a context-specific threshold relative to throughput and quality gains.

Derivation: TCE supports the logic of amortizing fixed deployment costs over larger case volumes, but clinical AI systems also incur fixed and recurring control costs. Scale effects therefore depend on whether privacy review, audit requirements, retraining, and validation obligations remain proportionate as deployment expands. Candidate outcomes include cost-per-eligible-patient, throughput under fixed staffing, and time-to-screening completion at different deployment scales.

#### Proposition 6

Proposition 6 (collaboration design → mediated organizational value): The impact of LLM adoption on recruitment outcomes is mediated by the collaboration design such that the same underlying model can produce different operational, equity, and economic consequences under different oversight and escalation configurations.

Derivation: This proposition integrates both STS and TCE. Poorly designed collaboration structures may suppress the value of strong model outputs or amplify bias and rework costs, whereas well-designed structures can translate the same technical capability into more stable organizational performance. Candidate outcomes include team-level variability in concordance, throughput, cost-per-case, and equity-sensitive differences in recruitment recommendations [11].

To make the propositions more inspectable and empirically actionable, Table 1 summarizes the focal mechanism, likely outcomes, and major moderators associated with each proposition family. The evaluation roadmap in the *Discussion* section then translates these proposition-level expectations into feasible study designs.

**Table 1.** Operationalization of LECRA^a^ propositions for future empirical testing.

Proposition	Core mechanism	Illustrative outcomes	Key moderators
P1	Higher data complexity increases the relative value of flexible language-based processing when records remain sufficiently accessible	Screening cycle time, criterion-level concordance, disagreement rate	Data completeness, access constraints, documentation inconsistency
P2	Net information-processing gain depends on model configuration, retrieval design, and privacy-control fit	Reviewer handling time, escalation frequency, operating cost per case	Cloud versus local architecture, retrieval design, audit requirements
P3	Hybrid workflows outperform pure manual or pure automation when rationales and override rights are well designed	Eligibility concordance, cycle time, reviewer confidence	Rationale quality, oversight design, workflow choreography
P4	High ambiguity and weak calibration increase hidden verification burden and alert fatigue under stronger automation	Override rate, correction burden, disagreement resolution time	Case ambiguity, hallucination sensitivity, alert volume
P5	Scale improves economics only when governance and validation costs remain proportionate	Cost-per-eligible-patient, throughput under fixed staffing	Audit burden, validation cost, compute cost trajectory
P6	Collaboration design mediates whether the same model produces stable organizational value	Between-team variability, throughput, equity-sensitive recommendation differences	Escalation rules, trust calibration, organizational redesign capacity

a LECRA: LLM-Embedded Clinical Recruitment Architecture.

## Discussion

### Principal Results

This study develops LECRA as a theory-grounded framework for analyzing LLM-enabled clinical trial recruitment as a sociotechnical and economic system rather than as a stand-alone matching task. The revised framework identifies 4 interdependent dimensions—data complexity, model configuration and processing, human-AI collaboration, and economic and governance consequences—and argues that recruitment outcomes emerge from their interaction rather than from model accuracy alone.

The central implication is that technical promise and organizational value should not be conflated. LECRA shifts the analytical question from whether an LLM can match patients with trials under benchmark conditions to when a particular configuration of model, workflow, and oversight can create a sustainable value under privacy, accountability, and trust constraints. This reframing is intended to make the framework more realistic for health care deployment and more testable for future research.

### Comparison With Prior Work

LECRA complements, rather than replaces, existing bodies of work. Recruitment-specific technical studies are highly informative about component-level matching performance, but they typically provide limited guidance on how those gains interact with workflow design, governance burden, and downstream organizational value. LECRA addresses this gap by linking technical outputs to sociotechnical and economic mechanisms in a recruitment-specific setting.

Compared with generic governance or trustworthy-AI frameworks, LECRA is narrower in scope but more explicit about workflow mechanisms. Frameworks, such as TRIAD [15], emphasize the need for safety, accountability, and real-world value, but they do not specify in detail how recruitment-specific data complexity, human review intensity, and scale conditions jointly shape operational outcomes. LECRA contributes by making those relationships proposition-ready.

The revised paper also positions LECRA against the broader evolution from dialogue-centric LLM use toward more integrated clinical AI and agent-like systems. LECRA remains focused on recruitment, but it now acknowledges that different model forms and system configurations can materially alter both technical and economic consequences.

Table 2 summarizes how LECRA is positioned relative to adjacent framework types.

**Table 2.** Positioning of LECRA^a^ relative to adjacent framework types.

Framework type	Primary unit of analysis	Treatment of human oversight	Treatment of economic logic	Main analytical output
Technical patient-trial matching studies	Model or pipeline performance	Usually downstream implementation issue	Rarely explicit	Benchmark or component-level comparison
Generic trustworthy AI^b^ or governance frameworks	Principles, controls, and deployment safeguards	High-level governance requirement	Usually implicit or secondary	Normative guidance
Broader clinical generative AI architectures	Clinical AI system design	Important but often not recruitment-specific	Variable, often not recruitment-centered	System-level architectural framing
LECRA	Recruitment workflow as sociotechnical transaction system	Core mediating mechanism with role, override, and escalation logic	Explicit through transaction-cost and governance-overhead reasoning	Recruitment-specific framework plus testable propositions

a LECRA: LLM-Embedded Clinical Recruitment Architecture.

b AI: artificial intelligence.

### Practical Implications

For organizations considering deployment, the practical implication is not simply to adopt the most capable model available. The stronger recommendation is to stage deployment around workflow fit, explanation design, and governance readiness. Human review should be treated as a configurable control mechanism rather than a temporary concession to imperfect technology. Similarly, apparent early efficiency gains should be interpreted alongside reviewer burden, auditability, and patient-facing trust considerations.

A phased rollout strategy remains sensible, but the revised framework suggests selecting pilots not only by volume but also by observability and governance tractability. High-volume use cases may still be poor candidates if documentation quality is unstable, privacy constraints are severe, or disagreement resolution is difficult to standardize. In practice, organizations should define escalation thresholds, rationale requirements, and stop rules before scaling model use.

A further implication is that implementation should be organized around explicit role allocation rather than a generic notion of “human oversight.” In a typical workflow, model outputs may support initial screening, but eligibility adjudication, discrepancy resolution, patient-facing communication, and governance review do not necessarily belong to the same actor. Clarifying decision rights at each stage can reduce duplication, make accountability visible, and help distinguish whether weak performance reflects model limitations, workflow design failures, or governance bottlenecks.

A related practical issue is the interface layer through which collaboration occurs. In deployed settings, LLM outputs are likely to be embedded in electronic health record views, recruitment dashboards, prescreening queues, or patient-facing intake interfaces rather than being presented as stand-alone model predictions. Interface design can therefore affect adoption, trust calibration, error correction, and escalation behavior. The present framework does not prescribe a specific user-interface design, but it treats integration with clinical workflow surfaces as an important direction for future implementation research.

Table 3 summarizes the illustrative workflow roles and decision rights that can guide LECRA-informed deployment.

**Table 3.** Illustrative workflow roles and decision rights in LECRA^a^-guided deployment.

Workflow component	Illustrative primary role	Decision right	Traceable output
Initial eligibility screening	Model-assisted coordinator or research staff	Flag candidate as likely eligible, uncertain, or likely ineligible	Structured rationale plus confidence/uncertainty note
Clinical adjudication of ambiguous cases	Clinician or qualified trial reviewer	Confirm, reject, or escalate model-supported screening outcome	Documented adjudication decision
Discrepancy resolution	Reviewer lead or multidisciplinary review point	Resolve conflict between model output and human interpretation	Escalation note and resolution record
Patient-facing communication	Recruitment coordinator or authorized clinical staff	Approve whether and how AI^b^-assisted messaging is used	Approved outreach text or contact decision

a LECRA: LLM-Embedded Clinical Recruitment Architecture.

b AI: artificial intelligence.

### Assumptions and Boundary Conditions

LECRA is most applicable under several baseline conditions: some minimum level of digital data availability, sufficient organizational capacity to redesign workflow interfaces, at least minimal governance capability, and an operating environment that is not so unstable that local practices change faster than the system can be adapted. These assumptions are important because the framework is intended to explain conditional value creation rather than universal model superiority.

When these assumptions are violated, expected benefits may weaken or reverse. Poor data quality can erase the value of more flexible model reasoning. Limited redesign capacity can convert hybrid review into duplicated work. Weak governance may allow technically impressive systems to create hidden compliance or trust costs. These boundary conditions should therefore be treated as substantive analytical features rather than background caveats.

### Evaluation Roadmap

The analytical structure is conceptual, but it is designed to be testable. A first empirical route would be retrospective workflow mapping that links model use, reviewer behavior, and case characteristics to cycle-time and concordance outcomes. A second route would be a prospective hybrid human-AI pilot in which rationale visibility, override authority, and escalation rules are deliberately specified. A third route would be a quasi-experimental multisite comparison, including approaches such as propensity-score-adjusted observational analysis [21], examining whether similar models produce different returns under different governance and workflow conditions.

Across these designs, suitable primary outcomes include screening cycle time, eligibility concordance with expert adjudication, throughput under fixed staffing, and cost per eligible patient. To reflect the framework’s acknowledgment of patient-facing pathways, secondary outcomes may also include consent conversion, refusal patterns after outreach, equity-sensitive differences in recommendation or contact rates, and documented concerns about the acceptability of AI-assisted communication. More abstract framework variables can also be operationalized with observable workflow measures. Governance overhead may be proxied by the number of approval steps, policy-review events, audit-log volume, privacy-review actions, or required model documentation tasks. Oversight burden may be measured through reviewer time on task, number of manual checks per case, escalation frequency, override rates, or discrepancy-resolution time. Verification burden may be captured by source-document verification counts, missing-document requests, reviewer queries, or time spent reconciling AI-generated summaries with original records. Important moderators include protocol complexity, data completeness, interoperability maturity, model architecture, governance intensity, and the degree of patient-facing automation. This roadmap is intended to answer the validation critique by making proposition testing executable without overstating what the present conceptual paper can claim.

### Limitations

First, this paper is conceptual and does not provide direct empirical validation. That limitation is partly mitigated by grounding the framework in published evidence, specifying mechanism-based propositions, and outlining a concrete evaluation roadmap. However, the propositions still require field testing before stronger causal or predictive claims can be made.

Second, the economic logic remains analytical rather than being estimated from field cost data. The revision strengthens this by incorporating governance, validation, and compliance overhead, but precise cost thresholds will vary across organizations and jurisdictions. Third, the framework necessarily simplifies legal and regulatory heterogeneity across contexts; although privacy and accountability constraints are now more explicit, future research should map LECRA against specific regulatory environments in greater detail. Fourth, the present model foregrounds recruitment workflows and does not extend fully to all downstream trial operations, such as retention, monitoring, or post-enrollment coordination. These limitations suggest a staged research agenda rather than a universal implementation prescription.

### Conclusions

LLMs are unlikely to resolve the clinical trial recruitment bottleneck through technical improvement alone. LECRA argues instead that the value emerges when model capability is aligned with data conditions, human oversight, and governance-economic constraints. By clarifying these mechanisms, boundary conditions, and future test paths, the framework is intended to support more realistic empirical research and more defensible implementation decisions in AI-enabled clinical recruitment.

## Abbreviations

AI: artificial intelligence
HL7 FHIR: Health Level Seven Fast Healthcare Interoperability Resources
LECRA: LLM-Embedded Clinical Recruitment Architecture
LLM: large language model
OMOP: Observational Medical Outcomes Partnership
SNOMED CT: Systematized Nomenclature of Medicine—Clinical Terms
STS: sociotechnical systems
TCE: transaction cost economics

## References

[1] Tang C, Sherman SI, Price M (2017). Clinical trial characteristics and barriers to participant accrual: the MD Anderson Cancer Center experience over 30 years, a historical foundation for trial improvement. Clin Cancer Res.

[2] Jin Q, Wang Z, Floudas CS (2024). Matching patients to clinical trials with large language models. Nat Commun.

[3] Rybinski M, Kusa W, Karimi S, Hanbury A (2024). Learning to match patients to clinical trials using large language models. J Biomed Inform.

[4] Rahmanian M, Fakhrahmad SM, Mousavi SZ (2025). Towards efficient patient recruitment for clinical trials: application of a prompt-based learning model. Healthc Inform Res.

[5] Zihang C, Liang L, Qianmin S, Gaoyi C, Jihan H, Ying L (2025). Enhanced pre-recruitment framework for clinical trial questionnaires through the integration of large language models and knowledge graphs. Sci Rep.

[6] Chen H, Li X, He X (2025). Enhancing patient-trial matching with large language models: a scoping review of emerging applications and approaches. JCO Clin Cancer Inform.

[7] Sharif MT, Rehman A (2025). Systematic literature review on clinical trial eligibility matching. arXiv.

[8] Beattie J, Neufeld S, Yang D (2024). Utilizing large language models for enhanced clinical trial matching: a study on automation in patient screening. Cureus.

[9] Gao M, Varshney A, Chen S (2025). The use of large language models to enhance cancer clinical trial educational materials. JNCI Cancer Spectr.

[10] Jin L, Ong JCL, Elangovan K (2025). Large language models in randomized controlled trials design: observational study. J Med Internet Res.

[11] Liu P, Zhang J, Chen S, Chen S (2025). Human-AI teaming in healthcare: 1 + 1 > 2?. NPJ Artif Intell.

[12] Parikh RB, Guido M, Girard A (2024). Human-AI teams to improve accuracy and timeliness of oncology trial prescreening: preplanned interim analysis of a randomized trial. J Clin Oncol.

[13] Hassan R, Nguyen N, Finserås SR, Adde L, Strümke I, Støen R (2025). Unlocking the black box: enhancing human-AI collaboration in high-stakes healthcare scenarios through explainable AI. Technol Forecast Soc Change.

[14] Khashu K (2026). Operationalizing trustworthy artificial intelligence in clinical and operational workflows. Front Digit Health.

[15] Li J, Zhou ZC, Wang ZC, Lv H (2025). Prioritizing human-AI collaboration in healthcare: the TRIAD framework for trustworthy governance, real-world, and integrated adaptive deployment. Mil Med Res.

[16] Trist EL, Bamforth KW (1951). Some social and psychological consequences of the longwall method of coal-getting: an examination of the psychological situation and defences of a work group in relation to the social structure and technological content of the work system. Hum Relat.

[17] Emery FE, Trist EL, Churchman CW, Verhulst M (1960). Management Sciences: Models and Techniques.

[18] Baxter G, Sommerville I (2011). Socio-technical systems: from design methods to systems engineering. Interact Comput.

[19] Williamson OE (1985). The Economic Institutions of Capitalism: Firms, Markets, Relational Contracting.

[20] Williamson OE (2005). The economics of governance. Am Econ Rev.

[21] Rosenbaum PR, Rubin DB (1983). The central role of the propensity score in observational studies for causal effects. Biometrika.

